# Metabolomics of Personalized Body Elements in Thai Traditional Medicine Response to Herbal Medicine for Body Elements Balancing in Healthy Volunteers

**DOI:** 10.1155/2023/6684263

**Published:** 2023-11-04

**Authors:** Manmas Vannabhum, Suthatip Mahajaroensiri, Saracha Pattanapholkornsakul, Athippat Tantiwongsekunakorn, Thapthep Thippayacharoentam, Pinpat Tripatara, Pravit Akarasereenont

**Affiliations:** ^1^Center of Applied Thai Traditional Medicine, Faculty of Medicine Siriraj Hospital, Mahidol University, Bangkok 10700, Thailand; ^2^Pharmacology Department, Faculty of Medicine Siriraj Hospital, Mahidol University, Bangkok 10700, Thailand; ^3^Siriraj Metabolomics and Phenomics Center, Faculty of Medicine Siriraj Hospital, Mahidol University, Bangkok 10700, Thailand

## Abstract

**Background:**

In Thai traditional medicine (TTM), the dominant body element called “Dhat Chao Ruean” (DCR) is an integral part in the diagnostic process of Thai traditional medicine. TTM practitioners usually use Thai herbal Benjakul formula (BKF) for adjusting and balancing the body elements. However, the effects of BKF on metabolism and individual response to it have not been studied yet.

**Methods:**

This study proposed to investigate the metabolic profiling in 24 volunteers categorized by their types of birth month DCR (bDCR) after the administration of BKF (450 mg, three tablets three times a day before meals) for seven days. Differences in metabolic profiling between bDCR groups were investigated by using liquid chromatography coupled with mass spectrometry for untargeted analysis, and in addition, the safety was assessed by testing the plasma biochemical level.

**Results:**

This study identified 57 biomarkers in positive ESI and 12 in negative ESI. Piperine was found in varying amount among the participants but it was the highest in the earth group. In addition, this study found that elemicin, phenylpropionic acid, ricinoleic acid, and *β*-sitosterol are important substances in a single herb of BKF. Regarding biochemical tests, the results indicated that BKF can decrease the lipid profile and it has no toxic effects on liver and kidney functions.

**Conclusion:**

The findings indicated that it is safe to use BKF which can help to improve health in chronic diseases by adjusting abnormality of the elements of the body. In addition, the information gathered from this study is valuable for further study in the field of Thai traditional medicine.

## 1. Introduction

Currently, Thai traditional medicine (TTM) plays an important role for the health and wellness of Thai population. According to the Ministry of Public Health of Thailand, up to 21.5% of Thai population [1] utilizes the different types of alternative medicine, such as Thai traditional medicine (TTM), traditional Chinese medicine (TCM), or Ayurvedic medicine. Globally, alternative-medicine options which employ traditional healing methods like herbal medication have also become extremely popular. In Thailand, TTM is extensively practiced and it includes the classification of the body's fundamental elements that are defined as Dhats. They are believed to regulate the chemical and physical functions in the human body. Dhat is divided into four major types as follows: earth (Dhat Din), water (Dhat Nam), wind (Dhat Lom), and fire (Dhat Fai) [1]. The feeling of wellbeing is associated with the right balance between these four elements. On the other hand, illness or discomfort can result from an imbalance of these elements where one may be deficient while the other is in excess. Dhat Din, the earth element, is generally quite powerful and it affects the other elements. The impairment is frequently associated with organ functions. Obesity, diarrhoea, and lymphatic symptoms are common in Dhat Nam, water-element impairment. Flatulence, dizziness, headache, and chronic discomfort are common symptoms of Dhat Lom, wind-element impairment, whereas constipation, allergies, and rash in Dhat Fai, fire-element impairment [1].

The intrinsic and extrinsic factors, such as a person's age, habits, behaviour, and seasons can interact with an individual's Dhat and result in illnesses. In addition to the aforementioned internal element composition, each individual has an inborn, dominating element known as “Dhat Chao Ruean” (DCR). DCR represents the four constituent elements in the human body. The varying severity and progression of what appears to be the same disease in individuals within a society are directly attributed to people having different distinct dominant components or DCR [1]. Unbalanced body elements can have an impact on other body elements, according to the TTM mechanism and cause of illness. DCR is crucial and it should be modified when an ailment becomes chronic. Chronic diseases, for example, chronic constipation, are associated with impairment in wind and water while headache from migraine with an excess of fire and obstruction of wind. After DCR is restored, the conditions that cause disease become the primary treatment goal.

In TTM scriptures such as Suppakunya, Kasai, and Chantasat, a Thai herb mixture known as Benjakul formula (BKF), is used in instances where the restoration of the balance of body elements is required. Its indications and uses were registered in the National List of Essential Medicines by the Ministry of Public Health in 2013 [2]. The formula is composed of five herbal components, namely, the fruit of *Piper retrofractum* Vahl., the root of *Piper sarmentosum* Roxb., *Plumbago indica* L., *Zingiber officinale* Roscoe, and the stem of *Piper interruptum* Opiz. (Supplementary Figure 3). Despite its widespread use and acceptance in Thailand, there is no clinical study which specifically describes BKF's biological activities to date.

Metabolomics is a tool used to assess the presence of drug metabolites in a given organism [3]. It is capable of identifying changes in biomarker molecules in the blood in response to a variety of medical interventions [4]. By combining TTM's classification of DCR and metabolomic changes after the consumption of BKF, we may begin to understand the biochemical profile which constitutes an individual's DCR. Therefore, the aim of this study was to investigate the metabolic profiling of individuals with different types of DCR before and after BKF administration by using the liquid chromatography technique and multidimensional pattern analysis.

## 2. Materials and Methods

### 2.1. Participants

This study was approved by the Siriraj Institutional Review Board (SIRB), Faculty of Medicine, Siriraj Hospital, Mahidol University 411/59 (EC1). Twenty four subjects were recruited from Siriraj Hospital and the written informed consents were obtained. The participants were screened before enrollment and only who fit the inclusion criteria were invited to participate. The enrollment criteria were age between 18 and 32 years and body mass index (BMI) between 18 and 24 kg/m^2^. Exclusion criteria were lactating or pregnant women, a history of allergic reactions to herbs or herbal remedies, a history of drug abuse, presence of underlying diseases necessitating the use of medications, a history of regular smoking and drinking alcohol more than 2 times per week, a history of blood transfusion within 3 months, or a history of medication, dietary supplements, or vitamins within 14 days prior to the start of the study. The participants who had taken foods or beverages containing the components of BKF, namely, *Piper retrofractum* Vah., *Piper sarmentosum* Roxb., *Piper interruptum* Opiz., *Plumbago indica* L., and *Zingiber officinale* Roscoe within 14 days before the study were also excluded, along with any patients with abnormal liver function and renal function tests. The participants were terminated during the study if they required drug administration or hospital admission or if they experienced adverse drug events or at the request of the physicians or the participant themselves. The participants who had abnormal physical examination or chemical blood tests or who failed follow-up appointment or contact were also terminated from the research. During the study, all participants were asked to record daily meal intakes and administration time in the case record form. The record form was brought back to the researcher in every visit. The record notes were checked to confirm the participants' correct drug administration times and whether they avoided foods containing ginger and pepper or not.

### 2.2. Subject Allocation

The participants were allocated to four groups, each group with 6 participants (3 males and 3 females). The bDCR was classified based on the month of birth [5] as follows: the fire group for December, January, and February; the wind group for March, April, and May; the water group for June, July, and August; and the earth group for September, October, and November.

### 2.3. Drug Administration

All participants received 70 BKF tablets (450 mg/tablet) in order to take 3 tablets 3 times per day, 30 minutes before meal, for seven days consecutively. The dosage and duration of BKF used in this study are in accordance with the recommendation by the National List of Essential Medicines and Herbal Medicines in 2013 [2]. In each visit, the leftover amount of BKF tablets was assessed.

### 2.4. Experimental Procedures and Blood Collection

**Figure 1:**
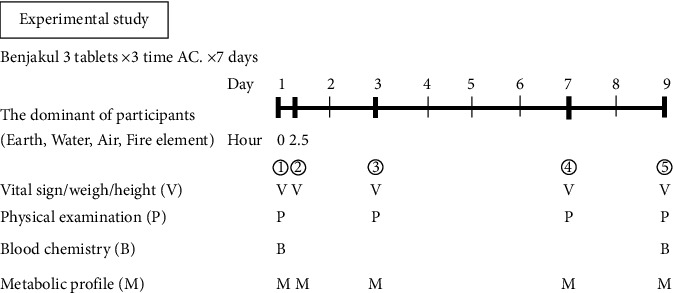
Experimental study.

On days 1, 3, 7, and 9, the blood samples were collected before and 2.5 hours after BKF administration for the metabolomics study, while the biochemical study was done on day 1 and 9 (see Figure 1). Biochemical study included (i) liver function test (AST, ALT, ALP, total bilirubin, albumin, and total protein), (ii) renal function test (BUN, serum creatinine, and uric acid), (iii) lipid profile (cholesterol, triglyceride, HDL, and LDL), (iv) fasting blood sugar level, and (v) complete blood count (RBC, WBC, hemoglobin, hematocrit, platelet count, mean corpuscular volume (MCV), mean corpuscular hemoglobin concentration (MCHC), and mean corpuscular hemoglobin).

### 2.5. Chemicals and Materials

For the metabolomics profiling study, methanol (LC-MS Optima grade) was purchased from Fisher Chemical, USA; caffeine (3-methyl-13C, 99%), cholic acid (2,2,4,4-D4, 98%), and L-phenylalanine (1-13C, 99%) (C6H5CH2CH (NH2) *∗* COOH) from Sigma-Aldrich (USA); formic acid (LC-MS grade) from Scharlau, Spain; the purified water was prepared by a Milli-Q water system (Millipore, France); and leucine enkephalin (C28H37N5O7) was purchased from Waters MS REF STDS: Q-Tof Product (Waters Corp., USA).

### 2.6. LC-QTOF Instruments and Conditions

Liquid chromatography (LC) was performed using the Acquity UPLC system (Waters Corp., USA) with an Acquity HSS T3 column (1.8 *μ*m, 2.1 × 100 mm) at 40°C constantly. The mobile phase consisted of 0.1% formic acid in purified water (A) and 0.1% formic acid in MeOH (B). The gradient program was set as follows: 100% A at 0 min, 100% B at 16 min, 100% A at 20 min, and maintaining 100% A at 24 min. For the injection analysis, 5 *μ*L of the sample volume was used with the flow rate of 400 *μ*L/min. The total running time for each sample was 24 minutes.

Nontargeted or qualitative analysis of metabolic profiling of the plasma samples was performed with a mass spectrometry (Waters® Xevo™ QTOF) equipped with an electrospray ionization (ESI) source. The analysis was operated in positive ion (ESI+) and negative ion (ESI−) with the MS^e^ analysis mode. Regarding source parameters, the setups were as follows: the collision energy of 4 V for low energy and 20 V for high, 3 kV of the electrospray capillary voltage, 40 V of the cone voltage, 150°C for the source temperature, and 500°C for the desolvation temperature. In addition, the cone and desolvation gas flow rate were set at 50 L/h and 1000 L/h, respectively. The full-scan mass range was set as 100–1200 Da for the continuum data type. During acquisition, the mass data underwent correction using an external reference (lock-spray) which comprised of leucine enkephalin solution at a flow rate of 10 *μ*L/min and scan injection every 20 sec, generating a reference ion for ESI + mode ([*M* + *H*] + with *m*/*z* 556.2771) and ESI− mode [*M* − *H*] ± with *m*/*z* 554.2615). Pooled QC samples were injected at the beginning of the run and at every 10^th^ sample in order to ensure accuracy and reproducibility throughout the MS analysis. All data collected were analyzed using MassLynx™ (V4.1) software.

### 2.7. Preparation of the Sample

Plasma preparation was used as in the previous study [6]. For the plasma samples' liquid extraction analysis, 100 *μ*L of each plasma sample was treated with 300 *μ*L of methanol and subsequently with 50 *μ*L of internal standard mix containing caffeine (trimethyl-13C_3_) and cholic acid (2,2,4,4-D_4_), of which the mass and concentration were known in order to monitor the efficiency and quantification of the extraction during analysis. The samples were then centrifuged at 15,800 rpm for 10 min at 4°C. The supernatants were collected, and 100 *μ*l and 30 *μ*l of the collections were used for analysis and a pooled QC sample, respectively. The specimens were analyzed in a random manner to prevent the batch effect.

### 2.8. Metabolites Identification

All chromatograms were analyzed in the multivariate analytical method using UNIFI Scientific Information System software. Principle component analysis (PCA) [7] and the orthogonal projections to latent structures-discriminant analysis (OPLS-DA) [8] were used for analyzing metabolic profiling. The S-plot was used to select biomarkers which are the metabolites that show significant differences between before and after BKF administration in the different groups of DCR. The selection conditions for biomarkers were as follows: a responding compound >50000 counts, variable importance in projection (VIP) value >1, and *p* value <0.05 [9, 10]. The selected biomarkers were identified by using online database such as ChemSpider, the Human Metabolome Database (HMDB), and KEGG pathway database. The setting of the identification included an error tolerance of less than 10 ppm and a retention time tolerance of 0.5 minutes. The dataset was identified by using traditional Chinese medicines (TCMs). The compound entry lists included chemical structure, molecular formula, and molecular weight in exact mass.

### 2.9. Statistical Analysis

All data are presented as the means and standard deviation (SD) and were statistically analyzed using 2-way ANOVA (PASW Statistics 18.0, SPSS). The level of statistical significance was set at a *p* value of less than 0.05. Differences in blood chemical among bDCR groups were assessed using both parametric and nonparametric tests, specifically one-way analysis of variance (ANOVA) and Kruskal–Wallis one-way ANOVA, respectively. All the graphs of statistical data analysis were created with GraphPad Prism version 5.00 for Windows, GraphPad Software (San Diego, California, USA).

## 3. Results and Discussion

### 3.1. The Characteristics of the Participants (*n* = 24)

There were no significant differences in the characteristics of subjects among the earth, water, wind, and fire groups at the baseline (Table 1).

### 3.2. Metabolites Profiling before and after BKF Administration

There were 120 blood samples collected from 24 participants both before and after BKF administration. The accuracy and reproducibility of all chromatogram data from LCMS-Q-TOF were ensured via continuous referencing of each sample using lock-spray and internal standards.

The PCA model was initially constructed from chromatographic data to identify clusters, groups, and outliers. The PCA results obtained before and after BKF administration were analyzed by employing both positive and negative ESIs, with R^2^Cum values of 74% and 79%, respectively. Consequently, the scatter plots for negative and positive ESI did not reveal any outliers (see Supplement Figures 1A and 1B). These plots demonstrated that metabolites from before and after BKF consumption were scattered into four main component classes. No significant differences were observed between the PCAs before and after BKF administration. However, an additional analysis using the OPLS-DA technique revealed detectable variations between the two groups (Supplementary Figures 1C and 1D).

**Figure 2:**
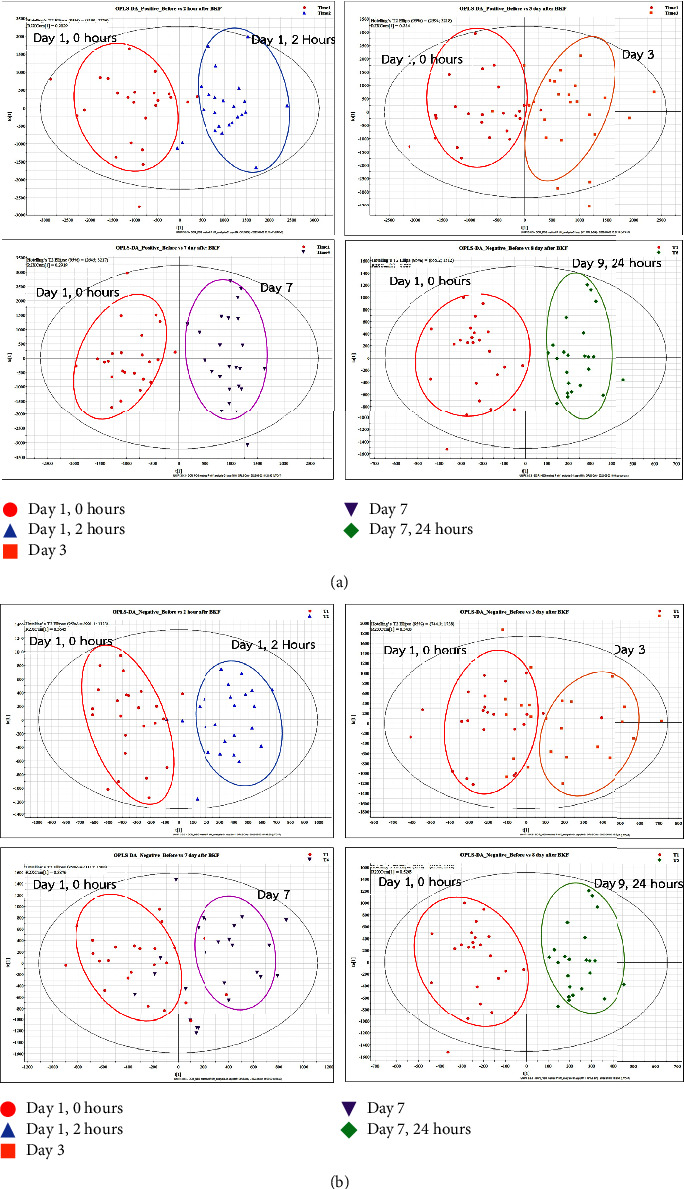
The comparison of before and after BKF administration: (a) OPLS-DA in positive ESI and (b) OPLS-DA in negative ESI.

When further analysis was done using the OPLS-DA technique, the analyses of the four subgroups demonstrated statistically significant results for all time intervals as shown in Supplementary Table 3 (Figure 2). The S-plots revealed variables in the extreme lower left and upper right quadrants as potential putative biomarkers for both before and after BKF treatment.

**Figure 3:**
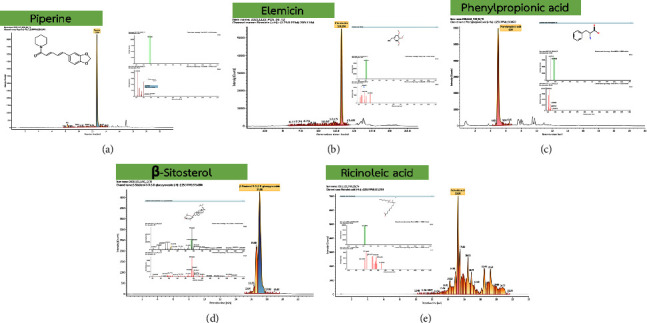
The compound of piperine (a), elemicin (b), phenylpropionic acid (c), and *β*-sitosterol (d), which were important substances in *Piper interruptum* Opiz. and Piper sarmentosum Roxb. The compound of *Zingiber officinale* Roscoe. was ricinoleic acid (e).

**Figure 4:**
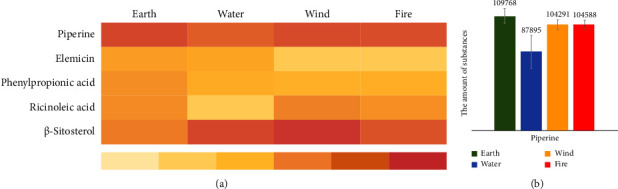
(a) The detected compounds in the plasma on day 7 after BKF administration in different DCR groups. (b) The mean quantity of piperine was found to be highest in the earth group.

A total of 57 biomarkers were found in positive ESI, while 12 were identified in negative ESI. The identification of these metabolites associated with BKF administration, along with their respective details, is presented in Supplementary Table 1 and Supplementary Figure 2. Furthermore, piperine was identified by its expected mass of 285.1364 Da and the retention time of 12.66 min (see Figure 3(a)). In addition, metabolites which are associated with individual herbs, such as ricinoleic acid in ginger, elemicin, phenylpropionic acid, and *β*-sitosterol, were identified as important substances in a single *Piper interruptum* Opiz. and *Piper sarmentosum* Roxb. (Figures 3(b)–3(e)). On day 7 after BKF administration, we used a heat map to represent the level of the active substances (Figure 4(a)) and the mean quantity of piperine was found to be maximum in the earth group (Figure 4(b)).

### 3.3. The Blood Chemical Analysis after BKF Administration

Differences in blood chemical results before and after taking BKF were analyzed using the paired sample *t*-test. The results showed that the lipid profile trended to decrease in values, such as cholesterol (187.17 ± 33.75 mg/dl), triglyceride (54.33 ± 27.07 mg/dl), and LDL (106.72 ± 34.20 mg/dl), demonstrating significant differences with *p* values of 0.006, 0.037, and 0.031, respectively. After BKF administration, aspartate transaminase (AST), a liver enzyme, decreased to 16.75 ± 3.97, showing significant differences compared to before BKF administration. The complete blood count (CBC) including hemoglobin, hematocrit, RBC count, lymphocytes, and eosinophils also decreased after taking BKF but remained within the normal range (Supplementary Table 2).

## 4. Discussion

“Dhat Chao Ruean” or the dominant body element is an important factor for TTM diagnosis and treatment. If a patient is diagnosed with a chronic disease, BKF can be used to improve imbalanced elements during the sickness [2]. This exploratory study was aimed to investigate metabolic profiling in the participants with different types of DCR before and after BKF administration. In addition, we examined blood clinical characteristics and conducted metabolomics study to provide evidence-based information for traditional knowledge from a scientific aspect.

Chemical compounds in plants (Photochemistry) refer to the compounds that plants produce through metabolic processes. The secondary metabolites are substances derived from the primary metabolites [11]. The biosynthesis process creates a variety of substances from three families: the phenolics, the alkaloids, and the steroids. In literature, elemicin, phenylpropionic acid, and *β*-sitosterol have already been tested for various properties, including the antineoplastic effect, an antimicrobial effect, inhibitory effect on human cancer cells proliferation [12], antioxidant activity, and immunomodulatory effect [13]. They were also confirmed to use their properties as elemental nourishing agents. In case studies of BKF recipes in treating stage 4 nonsmall cell lung cancer, the results showed the improvement in immunomodulation and quality of life with less systemic side effects [14]. Therefore, BKF would be useful for immunomodulation improvement, along with being a nourishing agent in Thai traditional medicine.

**Figure 5:**
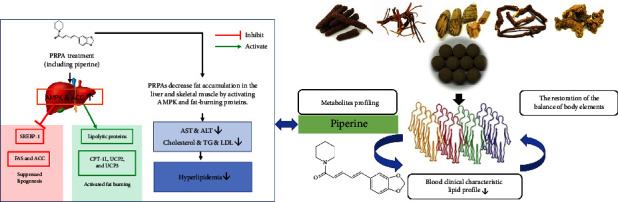
Piperidine alkaloids from *Piper retrofractum* Vahl. protect against high-fat diet-induced obesity by regulating lipid metabolism and activating AMP-activated protein kinase in mice [16], explaining the lipid-lowering mechanism of BKF in the volunteers.

Noncommunicable diseases (NCDs), also known as chronic diseases, usually develop and progress over long period, leading to premature morbidity, dysfunction, and a reduced quality of life. Among NCDs, the attributable mortality from overweight or obesity is remarkable [15]. According to the World Health Organization in 2020, approximately 2 billion adults are overweight. In this study, we found the decreasing trend in the lipid profile (cholesterol, triglyceride, and LDL) after BKF treatment. Interestingly, the compound piperine was found in the plasma after BKF treatment. Piperine is an alkaloid, which is an important active compound found in *Piper retrofractum* Vahl. *In vitro* and *in vivo* studies of piperidine alkaloids including piperine, pipernonaline, and dehydropipernonaline from *P. retrofractum* Vahl. (PRPAs) have already been conducted in high-fat diet (HFD)-induced obese mice. From the study, they were identified as antiobesity constituents through a peroxisome proliferator-activated receptor *δ* (PPAR*δ*) transactivation assay [16]. Therefore, BKF has been reported that it could become a new pharmacological agent to improve the lipid profile in obesity patients (Figure 5).

It is important to note that the metabolites which responded to BKF administration can significantly vary between different groups of bDCR. The difference of responded value compounds after the BKF administration could be different metabolites between the bDCR. Interestingly, *Piper interruptum* Opiz. is a medicinal herb with the earth element. Concordantly, piperine was detected prominently in the participants of earth bDCR, aligning with the Thai traditional scripture [1]. However, the results showed the variation in compound levels in each group of bDCR, possibly because of different time of maximum concentration and elimination. It is interesting how the DCR factor could contribute to different metabolites, warranting further experiments in the future.

In addition, the untargeted metabolomics is used to measure metabolites and serves as a bioinformatics tool to manage the dataset. The specific metabolites of interest will be confirmed through a targeted approach, which enhances accuracy and reliability using authentic standards [17]. The targeted metabolomics procedure ensures even greater precision. Therefore, in the next experiment, we will employ a targeted approach to confirm the results obtained from the untargeted analysis.

The comparison of the biochemical profile associated with BKF administration revealed a decrease in the liver function test [18] and lipid profile such as cholesterol, triglyceride, and LDL. A previous study about the safety of BKF, with participants taking 100 mg and 200 mg three times a day for 14 days, reported no abnormalities in all laboratory tests and the tests remained within normal values. Furthermore, the lipid profile exhibited a decreasing trend [19], consistent with our results. The aim of BKF administration is to balance or maintain the elements in the body and to improve the digestive system through the direct effect on the elements in the body. It would be interesting to study how BKF treatment in different types of bDCR could affect to change the biochemistry in the future.

The comparison of complete blood count (CBC) before and after BKF administration for all participants showed normal results, indicating BKF is safe to use for long duration. However, the limitation of the comparison of complete blood count (CBC) in the clinical experiment was the menstrual cycle in female participants, which could affect the blood level, intriguing the further study to handle this problem. Besides, another limitation of our study was sample size which was small, and hence, positive associations of the findings need to be confirmed with a large sample size.

DCR plays an important role in shaping the therapeutic options of chronic diseases which are defined broadly as conditions that persist for a year or longer, involving either continuous treatment or daily activities impairment or both. According to the TTM theory, the impact of these diseases varies with age and TTM classifies age into three categories as follows: children (0–16 years), middle (16–30 years), and elderly (beyond 30 years). This study specifically focused on the participants in their twenties, who are in the middle of their lives. According to the TTM theory, middle-aged disorders are related with the fire element, which coincide a higher prevalence of chronic diseases such as depression, hypertension, and insomnia. Conversely, wind-element-related disorders or symptoms such as dizziness, constipation, flatulence, and headache are common in people above the age of 30. However, the DCR of age above 30 should be addressed for the future study.

One limitation of this study is that the evaluation of DCR effects had to be performed exclusively in humans. Consequently, it was difficult to control for various factors, including demographics (e.g., gender, age, and body weight), the menstrual cycle in females, diet, and exercise [20], all of which could have influenced the metabolites observed. Therefore, it is possible that some of the observed metabolite variations during the experiment were attributable to these factors rather than the effects of BKF. For further studies, the study design should extend the washout period and more stringent control factors that can affect metabolites. In addition, increasing the sample size might help to reduce variables that may impact the experiments.

## 5. Conclusion

This is the first study investigating metabolomics profiling in the participants with different types of DCR before and after the BKF administration. We performed liquid chromatography coupled with the mass spectroscopy technique for untargeted analysis, and in addition, we evaluated safety through blood chemical characteristics analysis. Piperine, the main active compound in BKF, exhibited the highest mean quantity in the earth group. Regarding biochemical changes after BKF, a decreasing trend of the lipid profile, including cholesterol, triglyceride, and LDL, was observed, in turn attributing to the effect of hot-tasting drugs to increase the metabolic rate. Such effects align with the common use of BKF in promoting health, particularly in chronic disease management and elemental balance restoration in the body. This information provides scientific evidence for the rational utilization of BKF recipes in clinical practice.

However, the targeted analysis will still be needed to confirm this information. In addition, further studies should control the factors that can affect metabolites, such as diet, and consider increasing the sample size to reduce the variety of variables. In conclusion, this study provided valuable insights into the metabolites profile of bDCR after BKF, giving the data-based evidence for advancing research in Thai traditional medicine. The findings emphasize the importance of further investigations in this field and highlight its potential relevance for clinical practice.

## Figures and Tables

**Table 1:** The characteristics of the participants (*n* = 24).

Characteristics	Earth	Water	Wind	Fire
Gender (M/F)	3/3	3/3	3/3	3/3
Age (years)	26.33 ± 1.86	25.50 ± 2.17	25.67 ± 2.07	24.67 ± 2.50
BMI (kg/m^2^)	21.35 ± 2.03	21.80 ± 1.86	20.63 ± 2.22	20.27 ± 2.26

## Data Availability

The data used to support the findings of this study are available from the corresponding author upon reasonable request.

## Supplementary Materials

Supplementary Materials The following supplementary data form part of this article which further provide relevant information on some of the findings obtained from this study. Supplementary Table 1: the identification of metabolic profiling of participants after the BKF administration. Supplementary Table 2: the comparison of blood chemical before and after BKF administration. Supplementary Table 3: the OPLS-DA comparison of before and after BKF administration. Supplementary Figure 1: the comparison of PCA and OPLS-DA before and after BKF administration. Supplementary Figure 2: the biomarkers for discrimination comparing between before and after BKF administration that select the data presented in Figures 3 and 4. Supplementary Figure 3: the herbal compartments of BKF formula: (a) *Piper retrofractum* Vahl., (b) *Piper sarmentosum* Roxb., (c) *Plumbago indica* L., (d) *Zingiber officinale* Roscoe., and (e) *Piper interruptum* Opiz.
