# Supplementary material for: Metabolomics of Personalized Body Elements in Thai Traditional Medicine Response to Herbal Medicine for Body Elements Balancing in Healthy Volunteers
Source: Evid Based Complement Alternat Med. 2023 Nov 4;2023:6684263. doi: 10.1155/2023/6684263 (PMC10640159; doi:10.1155/2023/6684263)
Supplement: Supplementary Materials — The following supplementary data form part of this article which further provide relevant information on some of the findings obtained from this study. Supplementary Table 1: the identification of metabolic profiling of participants after the BKF administration. Supplementary Table 2: the comparison of blood chemical before and after BKF administration. Supplementary Table 3: the OPLS-DA comparison of before and after BKF administration. Supplementary Figure 1: the comparison of PCA and OPLS-DA before and after BKF administration. Supplementary Figure 2: the biomarkers for discrimination comparing between before and after BKF administration that select the data presented in Figures 3 and 4. Supplementary Figure 3: the herbal compartments of BKF formula: (a) Piper retrofractum Vahl., (b) Piper sarmentosum Roxb., (c) Plumbago indica L., (d) Zingiber officinale Roscoe., and (e) Piper interruptum Opiz. [file 6684263.f1.zip › Hindawi_Supplement file_DCR 2023 10 27.docx]

**Metabolomics of personalized Body Elements in Thai Traditional Medicine response to Herbal Medicine for Body elements balancing in Healthy Volunteers**

**Supplement table 1.**

The identification of metabolic profiling of participants after the BKF administration.

| **Component name** | **Molecular Formula** | **Molecular Weight (g/mol)** | **Expected mass** | **Observed mass** | **Observed (m/z)** | **Mass error (mDa)** | **Mass error (ppm)** |
| --- | --- | --- | --- | --- | --- | --- | --- |
|  |  |  | **(Da)** | **(Da)** |  |  |  |
| **Positive ESI** |  |  |  |  |  |  |  |
| (-)-Bornesitol | C7H14O6 | 194.18 | 194.08 | 194.08 | 195.09 | -0.11 | -0.58 |
| 1,2,3,6-Tetra-O-galloyl-β-D-glucopyranoside | C34H28O22 | 788.6 | 788.11 | 788.10 | 811.09 | -2.34 | -2.89 |
| 1,2,4,6-Tetra-O-galloyl-β-D-glucopyranoside | C34H28O22 | 788.6 | 788.11 | 788.10 | 811.09 | -2.41 | -2.97 |
| 16-O-Acetylisoiridogermanal | C46H78O6 | 516.36 | 516.38 | 516.38 | 517.38 | -4.00 | -7.73 |
| 1-Methyl-2-[(Z)-8-tetradecenyl]-4(1H)-quinolone | C21H29NO | 311.5 | 353.27 | 353.27 | 376.26 | -2.66 | -7.06 |
| 20(R)-Dammar-3β,6α,12β,20,25-pentaol | n/a | n/a | 494.40 | 494.39 | 517.38 | -4.28 | -8.28 |
| 23,27-Dihydroxypennogenin | n/a | n/a | 462.30 | 462.30 | 480.33 | 1.60 | 3.34 |
| 2H-1-Benzopyran-2-one | C9H14O2 | 154.21 | 146.04 | 146.04 | 169.03 | -0.99 | -5.86 |
| 2-Phenylethylisothio-cyanate | C9H9NS | 163.24 | 163.05 | 163.05 | 181.08 | 0.17 | 0.95 |
| 9,12-Dihydroxy-15-nonadecenoic acid | C19H36O4 | 328.5 | 328.26 | 328.26 | 351.25 | -2.69 | -7.66 |
| Adhyperforin | C36H54O4 | 550.82 | 550.40 | 550.40 | 568.43 | -5.40 | -9.49 |
| Anthranol | C14H10O | 194.23 | 194.07 | 194.07 | 195.08 | -1.57 | -8.07 |
| Bilirubin | C33H36N4O6 | 584.7 | 584.26 | 584.26 | 585.27 | -4.71 | -8.05 |
| Butyl isobutyl phthalate | C16H22O4 | 278.34 | 278.15 | 278.15 | 301.14 | 0.43 | 1.43 |
| Campesterol-β-D-glucoside | C34H58O6 | 562.8 | 562.42 | 562.42 | 563.43 | -3.90 | -6.92 |
| Casuariin | C34H24O22 | 784.5 | 784.08 | 784.08 | 807.07 | 1.35 | 1.67 |
| Chasmanine | C25H41NO6 | 451.6 | 451.29 | 451.29 | 469.33 | -1.37 | -2.92 |
| Coniferol | C10H12O3 | 180.2 | 180.08 | 180.08 | 181.08 | -1.62 | -8.95 |
| Cyclo-(Phe-Tyr) | C18H18N2O3 | 310.35 | 310.13 | 310.13 | 311.14 | -2.21 | -7.12 |
| Daturametelin E | n/a | n/a | 582.25 | 582.25 | 583.26 | 1.52 | 2.61 |
| Decumbesterone A | C29H46O7 | 506.7 | 506.32 | 506.32 | 524.36 | 0.05 | 0.10 |
| Dehydroanonaine | C17H13NO2 | 263.29 | 263.09 | 263.09 | 281.13 | -0.91 | -3.24 |
| Delbrusine | C27H43NO7 | 493.63 | 493.30 | 493.30 | 494.31 | 0.97 | 1.97 |
| Ecdysterone-20,22-monoacetonide | C30H48O7 | 520.7 | 520.34 | 520.34 | 521.34 | -4.07 | -7.80 |
| Elemicin | C12H16O3 | 208.26 | 208.11 | 208.11 | 209.12 | -0.84 | -4.00 |
| ent-16α,17-Hydroxy-19-kauranoic acid | C20H30O2 | 302.5 | 320.24 | 320.23 | 321.24 | -0.52 | -1.61 |
| Epianhydrobelachinal | C30H44O4 | 469.33 | 468.32 | 468.32 | 469.33 | -1.60 | -3.40 |
| Flavone | C15H10O2 | 222.24 | 222.07 | 222.07 | 240.10 | 0.07 | 0.27 |
| Galactose | C6H12O6 | 180.16 | 180.06 | 180.06 | 181.07 | -0.37 | -2.05 |
| Gentianidine | C9H9NO2 | 163.17 | 163.06 | 163.06 | 181.10 | 0.76 | 4.21 |
| Gentiatibetine | C9H11NO2 | 165.19 | 165.08 | 165.08 | 188.07 | -1.81 | -9.62 |
| Glycodeoxycholic acid | C26H43NO5 | 449.6 | 449.31 | 449.32 | 472.31 | 1.71 | 3.63 |
| Hordatine A | C28H38N8O4 | 550.7 | 550.30 | 550.30 | 568.34 | 1.23 | 2.17 |
| Isopropyl-p-benzalcohol | C7H8O | 108.14 | 136.09 | 136.09 | 154.12 | -1.02 | -6.63 |
| Juzirine | C17H15NO3 | 281.3 | 281.11 | 281.11 | 299.14 | 0.34 | 1.15 |
| Korsine N-oxide | n/a | n/a | 445.32 | 445.32 | 468.31 | 1.70 | 3.62 |
| Liriodenine | C17H9NO3 | 275.26 | 275.06 | 275.06 | 293.09 | -1.96 | -6.70 |
| Lycoctonine | C25H41NO7 | 467.6 | 467.29 | 467.29 | 468.30 | 0.94 | 2.00 |
| Meso-inositol | C6H12O6 | 180.16 | 180.06 | 180.06 | 181.07 | 0.25 | 1.40 |
| Methyl-β-D-frucopyranoside | C7H14O6 | 194.18 | 194.08 | 194.08 | 195.09 | -0.78 | -4.02 |
| N,N′-Dicarbazyl | n/a | n/a | 332.13 | 332.13 | 333.14 | -1.36 | -4.09 |
| Norbergenin | C13H14O9 | 314.24 | 314.06 | 314.06 | 315.07 | 0.71 | 2.26 |
| Palbinone | C22H30O4 | 358.5 | 358.21 | 358.22 | 376.25 | 0.67 | 1.79 |
| Papyriogenin D | C30H44O4 | 468.7 | 468.32 | 468.32 | 469.33 | 0.81 | 1.73 |
| Pedunculagin | C34H24O22 | 784.5 | 784.08 | 784.08 | 807.07 | 2.01 | 2.49 |
| Periplocoside N | C27H44O6 | 464.63 | 464.31 | 464.32 | 482.35 | 1.26 | 2.61 |
| Phenethyl ferulate | C18H18O4 | 298.33 | 298.12 | 298.12 | 299.13 | -1.23 | -4.13 |
| Phenylpropionic acid | C9H10O2 | 150.17 | 165.08 | 165.08 | 188.07 | -1.16 | -6.19 |
| Pingpeimine B | C27H45NO6 | 479.6 | 479.32 | 479.33 | 480.33 | 2.33 | 4.84 |
| Piperine | C17H19NO3 | 285.34 | 285.14 | 285.14 | 286.14 | 0.68 | 2.39 |
| Polyporusterone F | C28H46O5 | 462.7 | 462.33 | 462.33 | 463.34 | -2.13 | -4.60 |
| Rengyoxide | C8H14O3 | 158.2 | 158.09 | 158.09 | 181.08 | 0.49 | 2.72 |
| Ricinoleic acid | C18H34O3 | 298.5 | 298.25 | 298.25 | 321.24 | -1.27 | -3.94 |
| Thymine | C5H6N2O2 | 126.11 | 126.04 | 126.04 | 144.08 | 0.59 | 4.12 |
| tran-Ferulaldehyde | C₁₀H₁₀O₃ | 178.18 | 178.06 | 178.06 | 201.05 | 0.80 | 3.96 |
| Yakuchinone B | C20H22O3 | 310.39 | 310.16 | 310.15 | 333.14 | -3.15 | -9.45 |
| β-Sitosterol | C35H60O6 | 576.8 | 576.44 | 576.44 | 575.43 | 0.43 | 0.74 |
| **Nagative ESI** |  |  |  |  |  |  |  |
| 2β-Acetoxypterodontic acid | n/a | n/a | 292.17 | 292.17 | 291.16 | 0.77 | 2.66 |
| 3-O-α-L-Rhamnopyranosyl-(1→2)-α-L-arabinopyranosylgypsogenin | n/a | n/a | 748.44 | 748.45 | 747.44 | 5.71 | 7.65 |
| Auraptenol | C15H16O4 | 260.28 | 314.15 | 314.15 | 313.15 | 1.86 | 5.93 |
| Gramine | C11H14N2 | 174.24 | 174.12 | 174.11 | 173.11 | -0.84 | -4.83 |
| Hirsutine | C22H28N2O3 | 368.5 | 368.21 | 368.21 | 367.20 | 2.01 | 5.47 |
| Kalmanol | C20H34O6 | 370.5 | 370.24 | 370.24 | 369.23 | 0.48 | 1.31 |
| Marsdenoside C | C47H68O14 | 857.047 | 856.46 | 856.46 | 855.45 | 0.72 | 0.84 |
| Psammosilenins B | C45H62N8O9 | 859.022 | 858.46 | 858.47 | 857.46 | 5.62 | 6.56 |
| Quinatoside C | n/a | n/a | 720.41 | 720.41 | 719.40 | 1.69 | 2.34 |
| Taurodeoxycholic acid | C47H68O14 | 499.7 | 499.30 | 499.30 | 498.29 | -0.40 | -0.80 |
| Tenacissoside L | n/a | n/a | 832.48 | 832.49 | 831.48 | 5.74 | 6.91 |
| Yesanchinoside A | n/a | n/a | 858.50 | 858.49 | 857.49 | -3.91 | -4.56 |

**Supplement table 2.**

The comparison of blood chemical before and after BKF administration.

| **Characteristics** | | **Before** | | **After** | | **Ref Range** | | **p-value** | |
| --- | --- | --- | --- | --- | --- | --- | --- | --- | --- |
|  | | **(Mean±SD)** | | | |  | |  | |
| **Blood clinical** | |  | |  | |  | |  | |
| Glucose (mg/dl) | | 84.20±4.63 | | 83.21±6.12 | | 74-99 | | 0.359 | |
| BUN (mg/dl) | | 10.95±2.57 | | 11.48±2.16 | | 6-20 | | 0.311 | |
| Creatinine(mg/dl) | | 0.87±0.18 | | 0.86±0.19 | | 0.67-1.17 | | 0.601 | |
| eGFR (mL/min) | | 108.80±13.41 | | 109.18±14.18 | | MRR | | 0.818 | |
| Uric acid (mg/dl) | | 5.43±1.48 | | 5.36±1.34 | | 3.4-7.0 | | 0.536 | |
| Cholesterol (mg/dl) | | 194.45±37.93 | | 187.17±33.75 | | <200 | | 0.006* | |
| Triglyceride (mg/dl) | | 64.12±30.34 | | 54.33±27.07 | | <200 | | 0.037* | |
| HDL (mg/dl) | | 70.12±14.88 | | 69.59±14.52 | | >40 | | 0.611 | |
| LDL (mg/dl) | | 111.47±35.05 | | 106.72±34.20 | | <160 | | 0.031* | |
| Total protein (g/dl) | | 7.94±0.31 | | 7.84±0.37 | | 6.4-8.3 | | 0.209 | |
| Albumin (g/dl) | | 4.72±0.21 | | 4.64±0.23 | | 3.5-5.2 | | 0.171 | |
| Globulin (g/dl) | | 3.21±0.23 | | 3.19±0.31 | | 1.5-3.5 | | 0.734 | |
| Bilirubin (mg/dl) | | 0.64±0.28 | | 0.64±0.27 | | 0.0-1.2 | | 0.981 | |
| AST (U/L) | | 21.83±10.73 | | 16.75±3.97 | | 0-40 | | 0.007* | |
| ALT (U/L) | | 19.25±18.14 | | 15.96±7.84 | | 0-41 | | 0.195 | |
| ALP (U/L) | | 63.58±15.24 | | 62.87±15.79 | | 40-130 | | 0.463 | |
| **The complete blood count (CBC)** | |  | |  | |  | |  | |
| Hemoglobin (g/dl) | | 13.47±1.44 | | 13.18±1.66 | | 12.7-16.9 | | 0.017* | |
| Hematocrit (%) | | 42.58±3.96 | | 41.43±4.67 | | 40.3-51.9 | | 0.030* | |
| RBC count (10*6/µl) | | 4.94±0.55 | | 4.82±0.62 | | 4.2-6.1 | | 0.010* | |
| MCV (fl) | | 86.65±6.89 | | 86.47±7.40 | | 80.6-98.8 | | 0.723 | |
| MCH (pg) | | 27.40±2.49 | | 27.47±2.50 | | 25.8-33.1 | | 0.381 | |
| MCHC (g/dl) | | 31.64±1.29 | | 31.79±1.21 | | 30.8-34.6 | | 0.529 | |
| RDW (%) | | 13.58±1.65 | | 13.72±1.92 | | 11.9-14.5 | | 0.239 | |
| WBC (10*3*/ul) | | 6.20±2.28 | | 5.55±1.15 | | 4.5-11.3 | | 0.152 | |
| Platelet (10*3/ul) | | 268.33±49.19 | | 260.63±51.73 | | 160-356 | | 0.108 | |
| Absoluteneutrophils (10*3/ul) | | 3.12±0.70 | | 3.17±0.74 | | 2.1-7.2 | | 0.733 | |
| Neutrophils (%) | | 54.49±8.29 | | 57.57±8.69 | | 40.0-70.3 | | 0.035* | |
| Lymphocytes (%) | | 35.76±7.99 | | 32.69±7.89 | | 18.7-48.3 | | 0.041* | |
| Monocytes (%) | | 6.13±1.37 | | 6.55±1.12 | | 3.9-12.3 | | 0.097 | |
| Eosinophils (%) | | 2.96±2.34 | | 2.55±2.07 | | 0.8-9.2 | | 0.019* | |
| Basophils (%) | | .65±0.27 | | 0.65±0.25 | | 0.1-1.4 | | 0.777 | |

**P < 0.05* paired samples test

**Supplement table 3.**

The OPLS-DA comparison of before and after Benjakul administration

|  |  | The OPLS-DA comparison of before and after Benjakul administration | | |
| --- | --- | --- | --- | --- |
|  |  | R^2^X_Cum_ | R^2^YCum | Q^2^_Cum_ |
| before vs 2 hour | positive | 0.212 | 0.351 | 0.374 |
|  | negative | 0.365 | 0.405 | 0.338 |
| before vs day 3 | positive | 0.235 | 0.443 | 0.314 |
|  | negative | 0.344 | 0.373 | 0.357 |
| before vs day 7 | positive | 0.291 | 0.587 | 0.487 |
|  | negative | 0.387 | 0.609 | 0.485 |
| before vs day 9 | positive | 0.521 | 0.587 | 0.480 |
|  | negative | 0.527 | 0.337 | 0.465 |

**Supplement figure 1.**

The comparison of before and after BKF administration A) PCA in positive ESI B) PCA in negative ESI C) OPLS-DA in positive ESI D) OPLS-DA in negative ESI


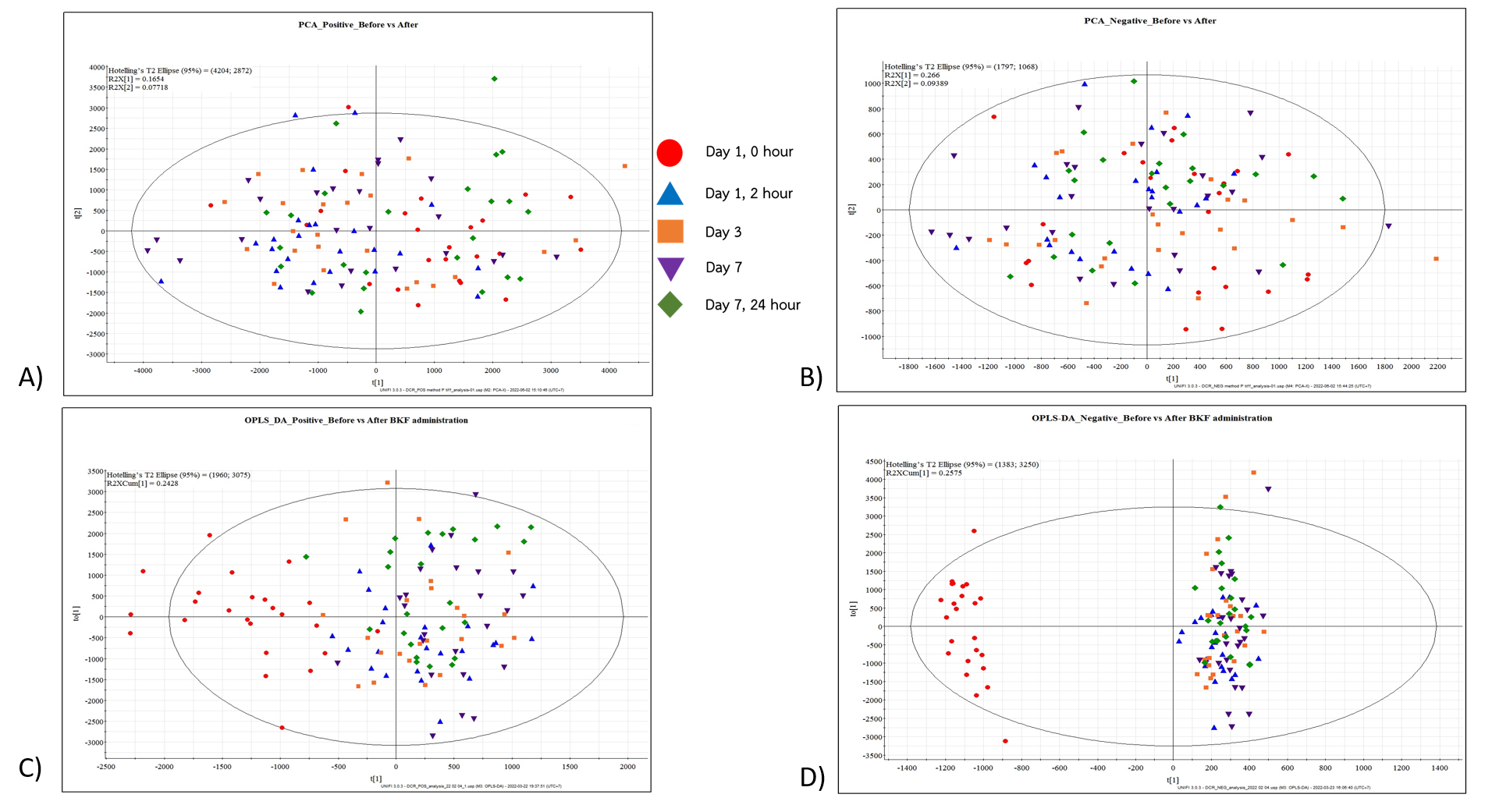


**Supplement figure 2.**

The biomarkers for discrimination comparing between before and after BKF administration were found 69 metabolites, 57 and 12 in positive and negative ESI, respectively.


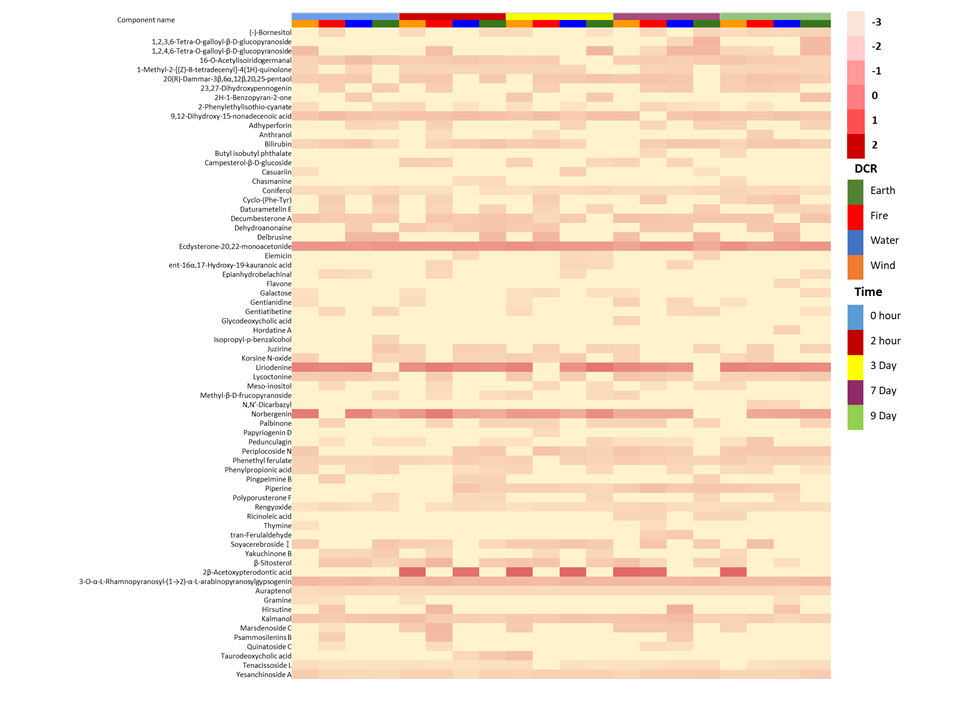


**Supplement figure 3.** The BKF formula is composed five herbal compartments a) *Piper retrofractum* Vahl, b) *Piper sarmentosum Roxb*., c) *Plumbago indica* L., d) *Zingiber officinale* Roscoe. and e) *Piper interruptum* Opiz.

**
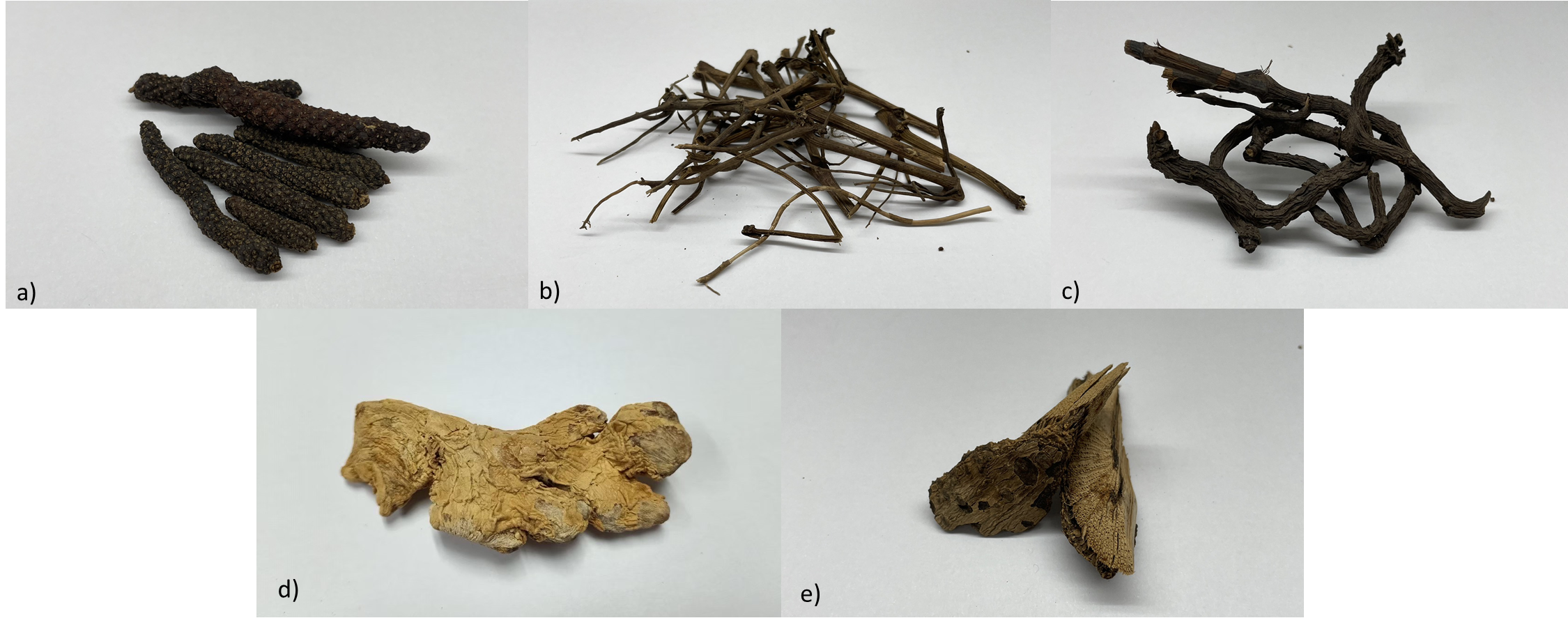
**
