# Supplementary material for: Metabolomics of Personalized Body Elements in Thai Traditional Medicine Response to Herbal Medicine for Body Elements Balancing in Healthy Volunteers
Source: Evid Based Complement Alternat Med. 2023 Nov 4;2023:6684263. doi: 10.1155/2023/6684263 (PMC10640159; doi:10.1155/2023/6684263)
Supplement: Supplementary Materials — The following supplementary data form part of this article which further provide relevant information on some of the findings obtained from this study. Supplementary Table 1: the identification of metabolic profiling of participants after the BKF administration. Supplementary Table 2: the comparison of blood chemical before and after BKF administration. Supplementary Table 3: the OPLS-DA comparison of before and after BKF administration. Supplementary Figure 1: the comparison of PCA and OPLS-DA before and after BKF administration. Supplementary Figure 2: the biomarkers for discrimination comparing between before and after BKF administration that select the data presented in Figures 3 and 4. Supplementary Figure 3: the herbal compartments of BKF formula: (a) Piper retrofractum Vahl., (b) Piper sarmentosum Roxb., (c) Plumbago indica L., (d) Zingiber officinale Roscoe., and (e) Piper interruptum Opiz. [file 6684263.f1.zip › Hindawi_Supplement file_DCR 2023 10 27.pdf]

Metabolomics of personalized Body Elements in Thai Traditional Medicine response to Herbal Medicine for Body elements balancing in Healthy Volunteers

Supplement table 1.

The identification of metabolic profiling of participants after the BKF administration.

Component name	Molecular Formula	Molecular Weight (g/mol)	Expected mass (Da)	Observed mass (Da)	Observed (m/z)	Mass error (mDa)	Mass error (ppm)
**Positive ESI**							
(-)-Bornesitol	C7H14O6	194.18	194.08	194.08	195.09	-0.11	-0.58
1,2,3,6-Tetra-O-galloyl-β-D-glucopyranoside	C34H28O22	788.6	788.11	788.10	811.09	-2.34	-2.89
1,2,4,6-Tetra-O-galloyl-β-D-glucopyranoside	C34H28O22	788.6	788.11	788.10	811.09	-2.41	-2.97
16-O-Acetylisoiridogermanal	C46H78O6	516.36	516.38	516.38	517.38	-4.00	-7.73
1-Methyl-2-[(Z)-8-tetradecenyl]-4(1H)-quinolone	C21H29NO	311.5	353.27	353.27	376.26	-2.66	-7.06
20(R)-Dammar-3β,6α,12β,20,25-pentaol	n/a	n/a	494.40	494.39	517.38	-4.28	-8.28
23,27-Dihydroxypennogenin	n/a	n/a	462.30	462.30	480.33	1.60	3.34
2H-1-Benzopyran-2-one	C9H14O2	154.21	146.04	146.04	169.03	-0.99	-5.86
2-Phenylethylisothio-cyanate	C9H9NS	163.24	163.05	163.05	181.08	0.17	0.95
9,12-Dihydroxy-15-nonadecenoic acid	C19H36O4	328.5	328.26	328.26	351.25	-2.69	-7.66
Adhyperforin	C36H54O4	550.82	550.40	550.40	568.43	-5.40	-9.49
Anthranol	C14H10O	194.23	194.07	194.07	195.08	-1.57	-8.07
Bilirubin	C33H36N4O6	584.7	584.26	584.26	585.27	-4.71	-8.05
Butyl isobutyl phthalate	C16H22O4	278.34	278.15	278.15	301.14	0.43	1.43

Component name	Molecular Formula	Molecular Weight (g/mol)	Expected mass (Da)	Observed mass (Da)	Observed (m/z)	Mass error (mDa)	Mass error (ppm)
Campesterol-β-D-glucoside	C34H58O6	562.8	562.42	562.42	563.43	-3.90	-6.92
Casuariin	C34H24O22	784.5	784.08	784.08	807.07	1.35	1.67
Chasmanine	C25H41NO6	451.6	451.29	451.29	469.33	-1.37	-2.92
Coniferol	C10H12O3	180.2	180.08	180.08	181.08	-1.62	-8.95
Cyclo-(Phe-Tyr)	C18H18N2O3	310.35	310.13	310.13	311.14	-2.21	-7.12
Daturametelin E	n/a	n/a	582.25	582.25	583.26	1.52	2.61
Decumbesterone A	C29H46O7	506.7	506.32	506.32	524.36	0.05	0.10
Dehydroanonaine	C17H13NO2	263.29	263.09	263.09	281.13	-0.91	-3.24
Delbrusine	C27H43NO7	493.63	493.30	493.30	494.31	0.97	1.97
Ecdysterone-20,22-monoacetonide	C30H48O7	520.7	520.34	520.34	521.34	-4.07	-7.80
Elemicin	C12H16O3	208.26	208.11	208.11	209.12	-0.84	-4.00
ent-16α,17-Hydroxy-19-kauranoic acid	C20H30O2	302.5	320.24	320.23	321.24	-0.52	-1.61
Epianhydrobelachinal	C30H44O4	469.33	468.32	468.32	469.33	-1.60	-3.40
Flavone	C15H10O2	222.24	222.07	222.07	240.10	0.07	0.27
Galactose	C6H12O6	180.16	180.06	180.06	181.07	-0.37	-2.05
Gentianidine	C9H9NO2	163.17	163.06	163.06	181.10	0.76	4.21
Gentiatibetine	C9H11NO2	165.19	165.08	165.08	188.07	-1.81	-9.62
Glycodeoxycholic acid	C26H43NO5	449.6	449.31	449.32	472.31	1.71	3.63
Hordatine A	C28H38N8O4	550.7	550.30	550.30	568.34	1.23	2.17
Isopropyl-p-benzalcohol	C7H8O	108.14	136.09	136.09	154.12	-1.02	-6.63
Juzirine	C17H15NO3	281.3	281.11	281.11	299.14	0.34	1.15
Korsine N-oxide	n/a	n/a	445.32	445.32	468.31	1.70	3.62

Component name	Molecular Formula	Molecular Weight (g/mol)	Expected mass (Da)	Observed mass (Da)	Observed (m/z)	Mass error (mDa)	Mass error (ppm)
Liriodenine	C17H9NO3	275.26	275.06	275.06	293.09	-1.96	-6.70
Lycoctonine	C25H41NO7	467.6	467.29	467.29	468.30	0.94	2.00
Meso-inositol	C6H12O6	180.16	180.06	180.06	181.07	0.25	1.40
Methyl-β-D-frucopyranoside	C7H14O6	194.18	194.08	194.08	195.09	-0.78	-4.02
N,N′-Dicarbazyl	n/a	n/a	332.13	332.13	333.14	-1.36	-4.09
Norbergenin	C13H14O9	314.24	314.06	314.06	315.07	0.71	2.26
Palbinone	C22H30O4	358.5	358.21	358.22	376.25	0.67	1.79
Papyriogenin D	C30H44O4	468.7	468.32	468.32	469.33	0.81	1.73
Pedunculagin	C34H24O22	784.5	784.08	784.08	807.07	2.01	2.49
Periplocoside N	C27H44O6	464.63	464.31	464.32	482.35	1.26	2.61
Phenethyl ferulate	C18H18O4	298.33	298.12	298.12	299.13	-1.23	-4.13
Phenylpropionic acid	C9H10O2	150.17	165.08	165.08	188.07	-1.16	-6.19
Pingpeimine B	C27H45NO6	479.6	479.32	479.33	480.33	2.33	4.84
Piperine	C17H19NO3	285.34	285.14	285.14	286.14	0.68	2.39
Polyporusterone F	C28H46O5	462.7	462.33	462.33	463.34	-2.13	-4.60
Rengyoxide	C8H14O3	158.2	158.09	158.09	181.08	0.49	2.72
Ricinoleic acid	C18H34O3	298.5	298.25	298.25	321.24	-1.27	-3.94
Thymine	C5H6N2O2	126.11	126.04	126.04	144.08	0.59	4.12
tran-Ferulaldehyde	$C_{10}H_{10}O_3$	178.18	178.06	178.06	201.05	0.80	3.96
Yakuchinone B	C20H22O3	310.39	310.16	310.15	333.14	-3.15	-9.45
β-Sitosterol	C35H60O6	576.8	576.44	576.44	575.43	0.43	0.74
**Nagative ESI**							

Component name	Molecular Formula	Molecular Weight (g/mol)	Expected mass (Da)	Observed mass (Da)	Observed (m/z)	Mass error (mDa)	Mass error (ppm)
2β-Acetoxypterodontic acid	n/a	n/a	292.17	292.17	291.16	0.77	2.66
3-O-α-L-Rhamnopyranosyl-(1→2)-α-L-arabinopyranosylgypsogenin	n/a	n/a	748.44	748.45	747.44	5.71	7.65
Auraptenol	C15H16O4	260.28	314.15	314.15	313.15	1.86	5.93
Gramine	C11H14N2	174.24	174.12	174.11	173.11	-0.84	-4.83
Hirsutine	C22H28N2O3	368.5	368.21	368.21	367.20	2.01	5.47
Kalmanol	C20H34O6	370.5	370.24	370.24	369.23	0.48	1.31
Marsdenoside C	C47H68O14	857.047	856.46	856.46	855.45	0.72	0.84
Psammosilenins B	C45H62N8O9	859.022	858.46	858.47	857.46	5.62	6.56
Quinatoside C	n/a	n/a	720.41	720.41	719.40	1.69	2.34
Taurodeoxycholic acid	C47H68O14	499.7	499.30	499.30	498.29	-0.40	-0.80
Tenacissoside L	n/a	n/a	832.48	832.49	831.48	5.74	6.91
Yesanchinoside A	n/a	n/a	858.50	858.49	857.49	-3.91	-4.56

Supplement table 2.

The comparison of blood chemical before and after BKF administration.

Characteristics	Before	After	Ref Range	p-value
	(Mean±SD)			
**Blood clinical**				
Glucose (mg/dl)	84.20±4.63	83.21±6.12	74-99	0.359
BUN (mg/dl)	10.95±2.57	11.48±2.16	6-20	0.311
Creatinine(mg/dl)	0.87±0.18	0.86±0.19	0.67-1.17	0.601

Characteristics	Before	After	Ref Range	p-value
	(Mean±SD)			
eGFR (mL/min)	108.80±13.41	109.18±14.18	MRR	0.818
Uric acid (mg/dl)	5.43±1.48	5.36±1.34	3.4-7.0	0.536
Cholesterol (mg/dl)	194.45±37.93	187.17±33.75	<200	0.006*
Triglyceride (mg/dl)	64.12±30.34	54.33±27.07	<200	0.037*
HDL (mg/dl)	70.12±14.88	69.59±14.52	>40	0.611
LDL (mg/dl)	111.47±35.05	106.72±34.20	<160	0.031*
Total protein (g/dl)	7.94±0.31	7.84±0.37	6.4-8.3	0.209
Albumin (g/dl)	4.72±0.21	4.64±0.23	3.5-5.2	0.171
Globulin (g/dl)	3.21±0.23	3.19±0.31	1.5-3.5	0.734
Bilirubin (mg/dl)	0.64±0.28	0.64±0.27	0.0-1.2	0.981
AST (U/L)	21.83±10.73	16.75±3.97	0-40	0.007*
ALT (U/L)	19.25±18.14	15.96±7.84	0-41	0.195
ALP (U/L)	63.58±15.24	62.87±15.79	40-130	0.463
**The complete blood count (CBC)**				
Hemoglobin (g/dl)	13.47±1.44	13.18±1.66	12.7-16.9	0.017*
Hematocrit (%)	42.58±3.96	41.43±4.67	40.3-51.9	0.030*
RBC count (10*6/µl)	4.94±0.55	4.82±0.62	4.2-6.1	0.010*
MCV (fl)	86.65±6.89	86.47±7.40	80.6-98.8	0.723
MCH (pg)	27.40±2.49	27.47±2.50	25.8-33.1	0.381
MCHC (g/dl)	31.64±1.29	31.79±1.21	30.8-34.6	0.529
RDW (%)	13.58±1.65	13.72±1.92	11.9-14.5	0.239
WBC (10*3*/ul)	6.20±2.28	5.55±1.15	4.5-11.3	0.152
Platelet (10*3/ul)	268.33±49.19	260.63±51.73	160-356	0.108

Characteristics	Before	After	Ref Range	p-value
	(Mean±SD)			
Absoluteneutrophils (10*3/ul)	3.12±0.70	3.17±0.74	2.1-7.2	0.733
Neutrophils (%)	54.49±8.29	57.57±8.69	40.0-70.3	0.035*
Lymphocytes (%)	35.76±7.99	32.69±7.89	18.7-48.3	0.041*
Monocytes (%)	6.13±1.37	6.55±1.12	3.9-12.3	0.097
Eosinophils (%)	2.96±2.34	2.55±2.07	0.8-9.2	0.019*
Basophils (%)	.65±0.27	0.65±0.25	0.1-1.4	0.777

*$P < 0.05$ paired samples test

Supplement table 3.

The OPLS-DA comparison of before and after Benjakul administration

		The OPLS-DA comparison of before and after Benjakul administration		
		R^2X_{Cum}	R^2YCum	Q^2_{Cum}
before vs 2 hour	positive	0.212	0.351	0.374
	negative	0.365	0.405	0.338
before vs day 3	positive	0.235	0.443	0.314
	negative	0.344	0.373	0.357
before vs day 7	positive	0.291	0.587	0.487
	negative	0.387	0.609	0.485
before vs day 9	positive	0.521	0.587	0.480
	negative	0.527	0.337	0.465

Supplement figure 1.

The comparison of before and after BKF administration A) PCA in positive ESI B) PCA in negative ESI C) OPLS-DA in positive ESI D) OPLS-DA in negative ESI

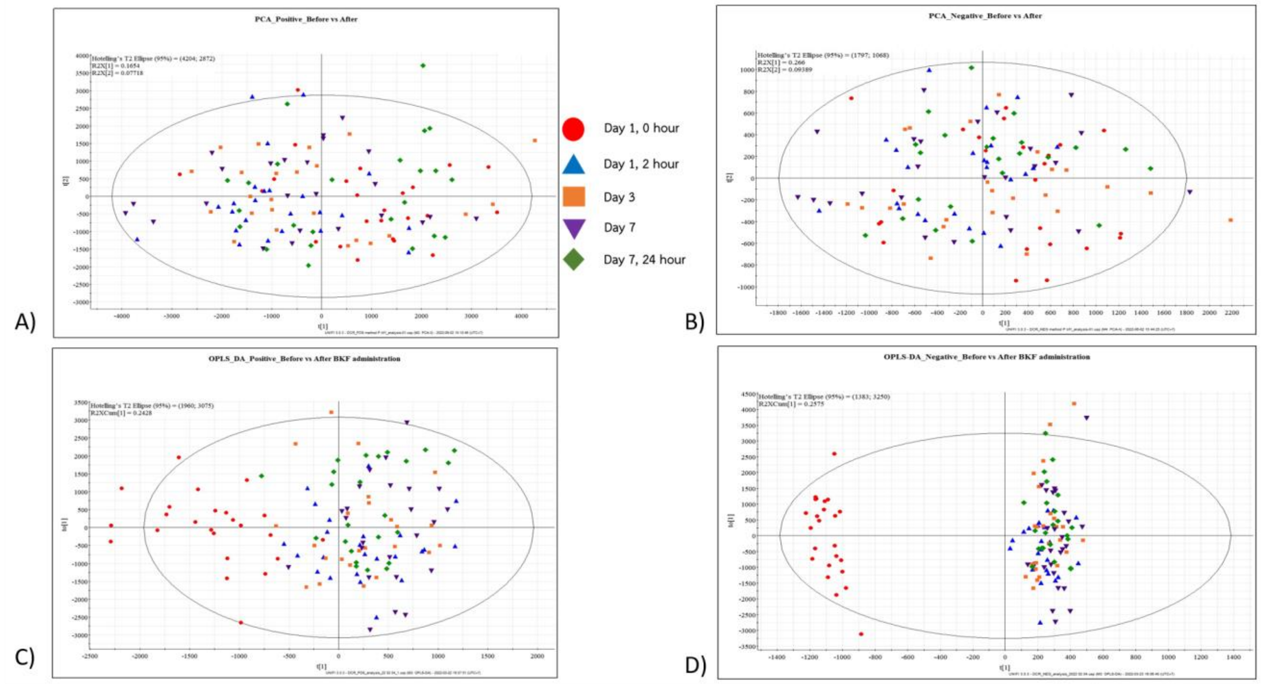

Supplement figure 2.

The biomarkers for discrimination comparing between before and after BKF administration were found 69 metabolites, 57 and 12 in positive and negative ESI, respectively.

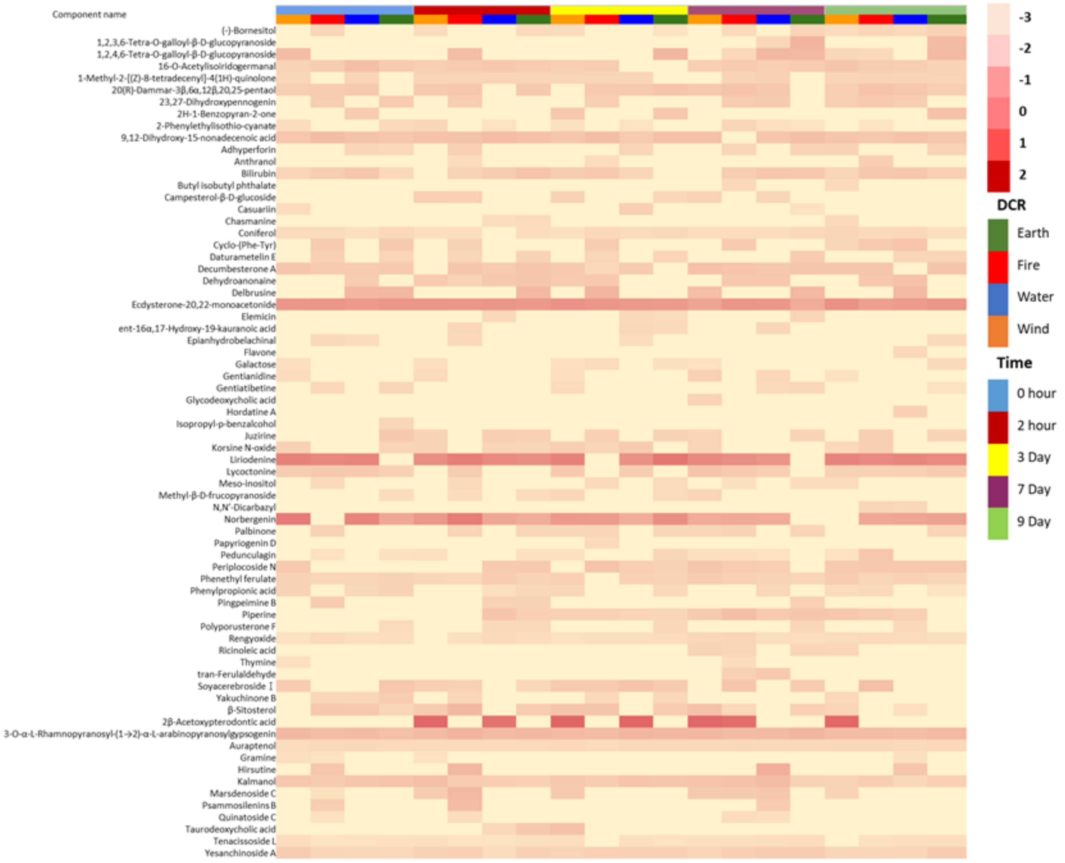

Supplement figure 3. The BKF formula is composed five herbal compartments a) *Piper retrofractum* Vahl, b) *Piper sarmentosum Roxb*., c) *Plumbago indica* L., d) *Zingiber officinale* Roscoe. and e) *Piper interruptum* Opiz.

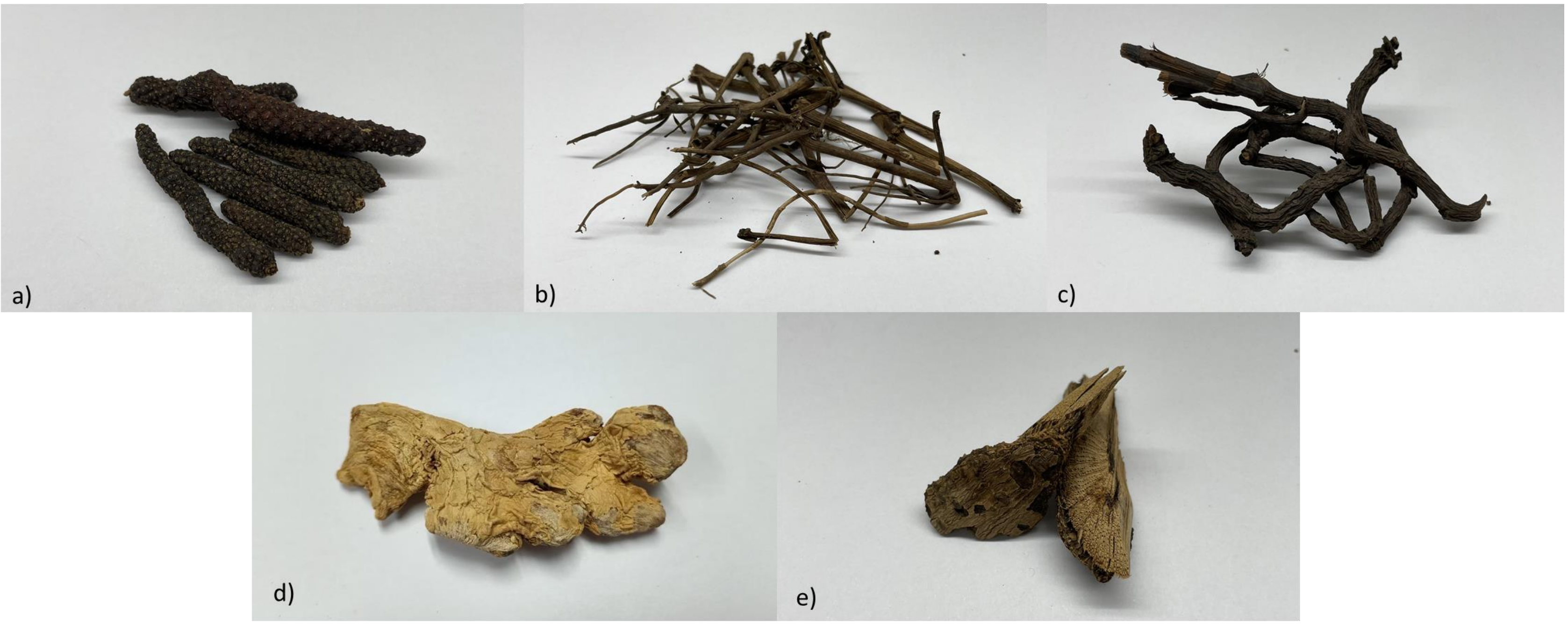
a)
b)
c)
d)
e)
